# Olfactory and cortical projections to bulbar and hippocampal adult-born neurons

**DOI:** 10.3389/fnana.2015.00004

**Published:** 2015-02-02

**Authors:** Carlos De La Rosa-Prieto, Miguel De Moya-Pinilla, Daniel Saiz-Sanchez, Isabel Ubeda-banon, Dulce M. Arzate, Alicia Flores-Cuadrado, Teresa Liberia, Carlos Crespo, Alino Martinez-Marcos

**Affiliations:** ^1^Neuroplasticity and Neurodegeneration Laboratory, Department of Medical Sciences, Ciudad Real Medical School, El Centro Regional de Investigaciones Biomédicas, Universidad de Castilla-La ManchaCiudad Real, Spain; ^2^Department of Cellular and Molecular Neurobiology, Neurobiology Institute, Universidad Nacional Autónoma de MéxicoQuerétaro, México; ^3^Departamento de Biología Celular, Facultad de Ciencias Biológicas, Universidad de ValenciaBurjassot, Spain

**Keywords:** adult neurogenesis, hippocampus, memory, olfaction, tract-tracing, synapse

## Abstract

New neurons are continually generated in the subependymal layer of the lateral ventricles and the subgranular zone of dentate gyrus during adulthood. In the subventricular zone, neuroblasts migrate a long distance to the olfactory bulb where they differentiate into granule or periglomerular interneurons. In the hippocampus, neuroblasts migrate a short distance from the subgranular zone to the granule cell layer of the dentate gyrus to become granule neurons. In addition to the short-distance inputs, bulbar interneurons receive long-distance centrifugal afferents from olfactory-recipient structures. Similarly, dentate granule cells receive differential inputs from the medial and lateral entorhinal cortices through the perforant pathway. Little is known concerning these new inputs on the adult-born cells. In this work, we have characterized afferent inputs to 21-day old newly-born neurons. Mice were intraperitoneally injected with bromodeoxyuridine. Two weeks later, rhodamine-labeled dextran-amine was injected into the anterior olfactory nucleus, olfactory tubercle, piriform cortex and lateral and medial entorhinal cortices. One week later, animals were perfused and immunofluorescences were carried out. The data show that projection neurons from the mentioned structures, establish putative synaptic contacts onto 21-day-old neurons in the olfactory bulb and dentate gyrus, in some cases even before they start to express specific subpopulation proteins. Long-distance afferents reach middle and outer one-third portions of the molecular layer of the dentate gyrus and granule and, interestingly, periglomerular layers of the olfactory bulb. In the olfactory bulb, these fibers appear to establish presumptive axo-somatic contacts onto newly-born granule and periglomerular cells.

## Introduction

The subventricular zone of the lateral ventricles (SVZ) and the subgranular zone (SGZ) of the dentate gyrus (DG) were described as the two main neurogenic niches of the adult mammalian forebrain (Altman, 1962; Altman and Das, 1965; Luskin, 1993; Lois and Alvarez-Buylla, 1994). Decades later, the functional significance of new neurons integrated into adult olfactory bulb (OB) and DG is only partially known (Aimone et al., 2010; Sahay et al., 2011; Sakamoto et al., 2014).

In the SVZ, neuroblasts migrate tangentially during about 7 days through the rostral migratory stream to the OB (Alvarez-Buylla and Garcia-Verdugo, 2002) following 2 major signals, structural (astrocytes, vasculature and extracellular matrix) and molecular (BDNF, Netrins, Slits, etc.) factors (Nguyen-Ba-Charvet et al., 2004; Whitman et al., 2009; Kaneko et al., 2010). There, they migrate radially to mature into granule (Petreanu and Alvarez-Buylla, 2002) or periglomerular (Carlen et al., 2002; Lledo et al., 2006) interneurons 15 (Petreanu and Alvarez-Buylla, 2002) and 28 (Belluzzi et al., 2003) days after birth, respectively. In the DG, neuroblasts migrate a short distance from the SGZ to the granule cell layer to become fully functional neurons after 4–28 days (Van Praag et al., 2002).

This differentiation and integration implies hodological and functional changes (Petreanu and Alvarez-Buylla, 2002; Lledo and Saghatelyan, 2005; Lepousez et al., 2013). Projections from bulbar mitral and tufted cells to olfactory-recipient areas in the basal telencephalon (Pro-Sistiaga et al., 2007; Martinez-Marcos, 2009) are reciprocated by centrifugal projections to the bulb arising from the anterior olfactory nucleus (AON), the olfactory tubercle (OT), the olfactory amygdala and the piriform (PIR) and lateral entorhinal (LEC) cortices (Mohedano-Moriano et al., 2012). Newly-born olfactory granule cells first receive intrinsic GABAergic synaptic connections (3–7 days after birth) and extrinsic glutamatergic inputs thereafter (Belluzzi et al., 2003; Carleton et al., 2003; Panzanelli et al., 2009; Lepousez et al., 2013). New neurons integrate into functional circuits in a “listen before talk” manner (Whitman and Greer, 2009). These centrifugal inputs and the ambient GABA signals may play an important role in maintenance and synaptic integration by regulation of dendritic growth of newly generated neurons in preexisting adult neuronal networks (Gascon et al., 2006; Mouret et al., 2009; Pallotto and Deprez, 2014). Interestingly, LEC send centrifugal inputs to the OB and also projects to the hippocampus.

Dentate granule cells receive, through the perforant path, massive afferents from the lateral (LEC) and medial (MEC) entorhinal cortices (Steward, 1976; Witter, 2007), which ends in the outer and middle one-thirds of the molecular layer of the DG, respectively (Ramón Y Cajal, 1893; Hjorth-Simonsen, 1972; Hjorth-Simonsen and Jeune, 1972). Newly-born dentate granule cells initially receive excitatory GABAergic inputs from local interneurons, and this becomes inhibitory only once the glutamatergic extrinsic input has established, 2 weeks after birth (Overstreet Wadiche et al., 2005; Toni et al., 2007). In the same manner, these inputs seem to play an essential role for cell survival, maturation and maintenance of new dentate granule cells in the hippocampal circuitry (Van Der Borght et al., 2005; Tashiro et al., 2006; Li et al., 2012).

Therefore, in the present work we have tried to characterize inputs arising from different structures onto 21 day-old adult-born granule cells in the OB and DG of adult mice. These data could help to understand the anatomical and physiological changes underlying the integration of neurogenic elements in the adult brain.

## Material and methods

### Experimental animals

Thirty adult (6 weeks) mice (C57BL/J) of both sexes (15/15) were obtained from Charles River (Barcelona, Spain) and maintained under controlled temperature and a 12:12 h light/dark cycle with food and water *ad libitum*. Experimental procedures were carried out according to guidelines of the Spanish (RD53/2013) and European (Directive 2010/63/EU) legislation of the protection of animals used for experimental purpose, and the Ethical Committee of Animal Research of the University of Castilla-La Mancha.

### Bromodeoxyuridine administration

BrdU (5-bromo-2′-deoxyuridine, Fluka, Madrid, Spain) administration included 4 ip doses (at 2-h intervals) of 10 mg/mL BrdU in phosphate-buffered saline (PBS, 0.15 M NaCl, 0.01 M sodium phosphate pH 7.4) totalizing a dose of 200 mg/kg in 1 day. This dose was employed following previous results in our laboratory to optimize labeling without increasing apoptosis (Martinez-Marcos et al., 2005; De La Rosa-Prieto et al., 2009a).

### Tracer injections and perfusion

Two weeks after BrdU administration, animals were anesthetized with a combined dose of ketamine hydrochloride (Ketolar, Parke-Davis, Madrid, Spain, 1.5 mL/kg, 75 mg/kg) and xylazine (Xilagesic, Calier, Barcelona, Spain, 0.5 mL/kg, 10 mg/kg). Under stereotaxic control, rhodamine-labeled dextran-amine (RDA, 10,000 mW, lysine fixable, Molecular Probes, Eugene, OR; 10% diluted in PBS) was ionophoretically injected (30–80 μm diameter tip; positive current pulses 7/7 s; 2–7 μA; 8–20 min) at the intended injection sites. Five experimental groups (*n* = 6, 3 M, F) totalizing *N* = 30 rodents were established according to coordinates relative to Bregma: AON (anterior 2.6; lateral 1; depth 2.7), OT (anterior 1.1; lateral 1.5; depth 4.7), PC (anterior 1.1; lateral 2.5; depth 4), LEC (posterior −4.36; lateral 4; depth 2), and MEC (posterior −4.5; lateral 3.3; depth 3) (Franklin and Paxinos, 2008). In all experimental groups (*n* = 6), a minimum of five cases included injections restricted at intended site and, therefore, comparable.

One week afterwards, animals were anesthetized (as above) and perfused with saline solution followed by 4% w/v paraformaldehyde fixative in phosphate buffer (0.1 M sodium phosphate pH 7.2). Brains were postfixed in 4% w/v paraformaldehyde, cryoprotected in 30% w/v sucrose, and sagittally (olfactory bulb) or frontally sectioned (50 μm) using a freezing sliding microtome (Microm HM450). Sections were consecutively collected into 96-well plates and maintained at 4°C in preserving solution (PBS containing 20% v/v glycerol and 30% v/v ethylene glycol) for further processing.

### Immunofluorescence procedures

Sections were rinsed overnight with Tris-buffered saline (TBS; 0.15 M NaCl, 0.05 M Tris, HCl pH 7.6), fixed with 4% w/v paraformaldehyde in phosphate buffer (PB; 0.1 M sodium phosphate pH 7.2) for 30 min at 37°C, rinsed in TBS (3 × 5 min), treated with 37% w/v HCl for 20 min at 37°C and then with pepsin 0.5 mg/mL in 37% w/v HCl for 20 min at 37°C. Sections were rinsed (3 × 10 min) and blocked with 10% normal donkey serum (Vector Laboratories, Burlingame, CA) in TBS for 30 min. Sections were then incubated overnight with mouse anti-BrdU (1:40, Dako, M0744, Glostrup, Denmark) or rat anti-BrdU (1:40; Santa Cruz, SC-70441, CA, USA) and rabbit anti-calretinin (CR, 1:500, Swant, Bellinzona, Switzerland), goat anti-doublecortin (DCX, 1:200, Santa Cruz, sc-8066, CA, USA) and rabbit anti-postsynaptic density-95 (PSD-95, 1:50, Santa Cruz, sc-28941, CA, USA) antibodies diluted in TBS with 1% v/v normal goat serum at 4°C. Antibodies specificity was assessed by using untreated animals and/or omitting primary antibodies. Sections were rinsed and incubated with Alexa 405 anti-mouse, Alexa 488 anti-mouse or anti-rat, Alexa 594 anti-rabbit and Alexa 647 anti-goat (1:200 in TBS with 2% of normal goat serum and 0.2% Triton X-100; Invitrogen, Eugene, OR), and counterstained using DAPI (1 μg/ml in TBS, Santa Cruz, SC-3598) for 5 min in the dark. All sections were mounted and coverslipped with PVA-DABCO solution (DABCO®).

### Analysis of labeled cells

Sections under study were analyzed using epifluorescence (Nikon eclipse Ti) and confocal microscopy (Zeiss LSM 710). Brightness, contrast and gamma of images were adjusted using Irfanview software and figures arranged and lettered using Canvas software.

## Results

### Injection sites

**Figure 1:**
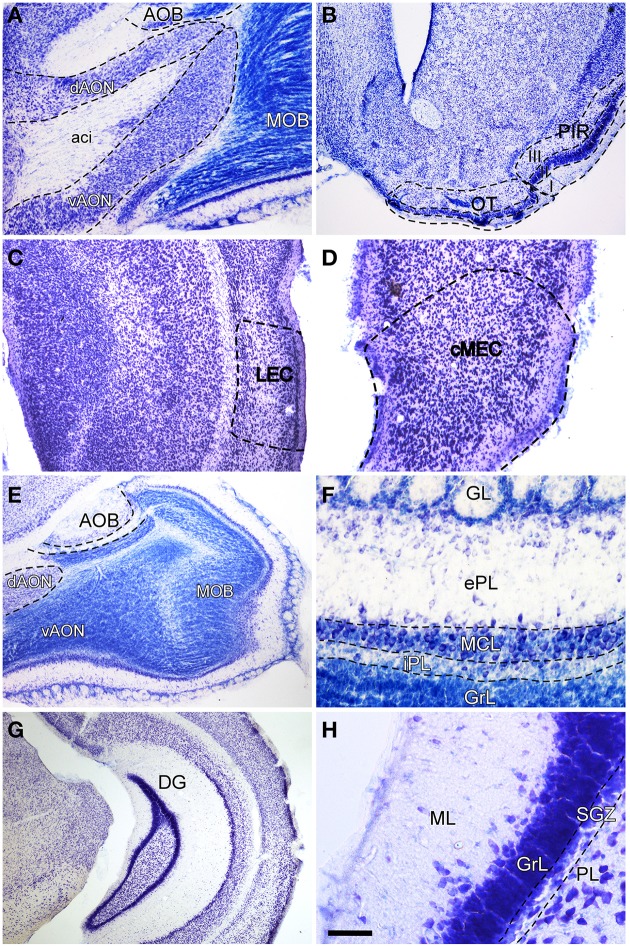
**Nissl-stained coronal (B–D,G,H) and sagittal (A,E,F) sections of olfactory bulb and dentate gyrus**. Aci, anterior commissure intrabulbar; AOB, accessory olfactory bulb; cMEC, caudo-medial entorhinal cortex; dAON, anterior olfactory nucleus dorsal; DG, dentate gyrus; ePL, external plexiform layer olfactory bulb; GL, glomerular layer olfactory bulb; GrL, granular layer; iPL, internal plexiform layer olfactory bulb; LEC, lateral entorhinal cortex; MCL, mitral cell layer; ML, molecular layer dentate gyrus; MOB, main olfactory bulbs; OT, olfactory tubercle; PIR, piriform cortex; PL, polymorph layer of the dentate gyrus; SGZ, subgranular zone; vAON, anterior olfactory nucleus ventral. Calibration bar for **(A)** 334 μm; **(B** y **G)** 500 μm; **(C** y **E)** 250 μm; **(D)** 167 μm; **(F)** 100 μm; **(H)** 50 μm.

In order to label afferent connections to the olfactory bulb arising from olfactory-recipient structures (Mohedano-Moriano et al., 2012) and to the dentate gyrus originating in the entorhinal cortex, injections were aimed at the AON (Figure 1A), OT (Figure 1B), PIR (Figure 1B), LEC (Figure 1C) and MEC (Figure 1D), respectively. The resulting anterograde labeling was analyzed in the different layers of the olfactory bulb (Figure 1E), particularly in the granule cell (GrL) and glomerular (GL) layers (Figure 1F) as well as in the caudoventral DG (Figure 1G), specifically in the molecular layer (Figure 1H).

**Figure 2:**
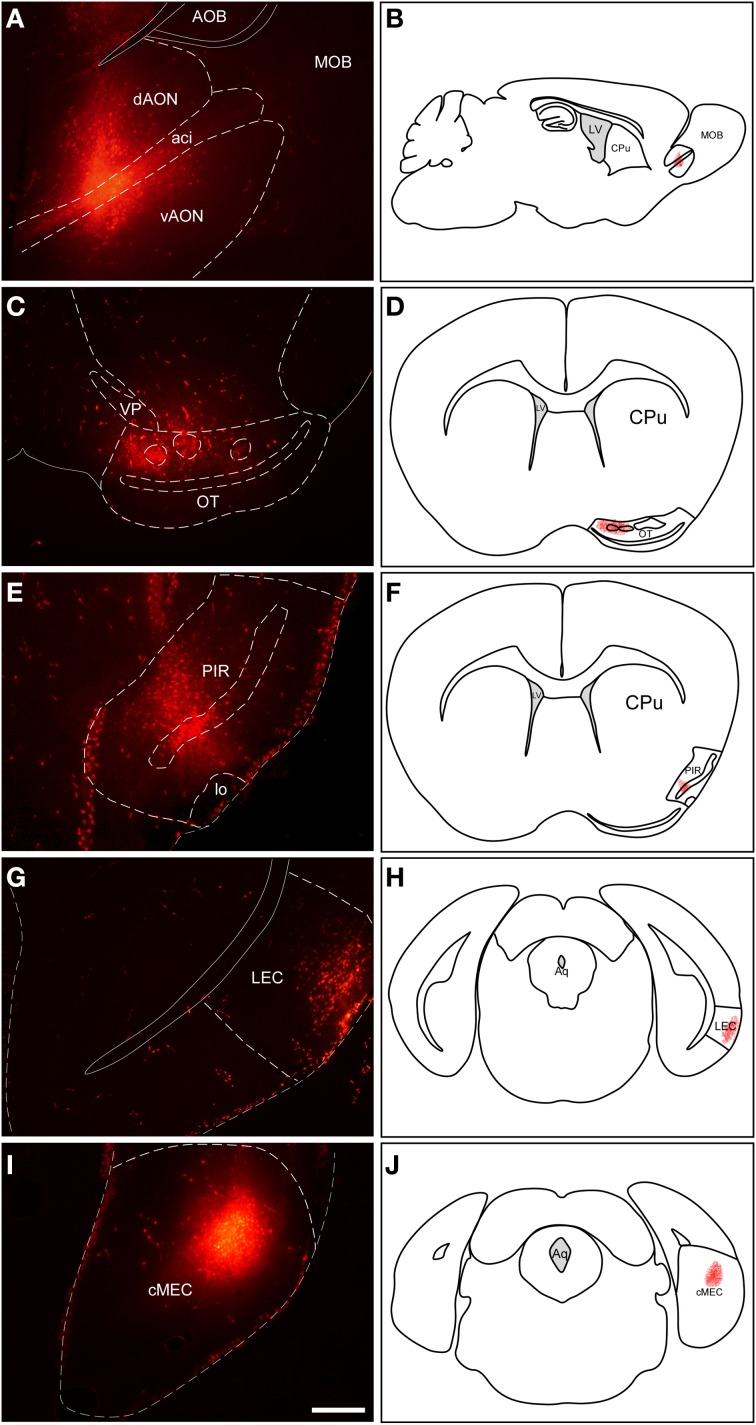
**Injection sites of rhodamine-labeled dextran-amine (RDA) tracer in anterior olfactory nucleus **(A,B)**, olfactory tubercle **(C,D)**, piriform cortex **(E,F)**, lateral entorhinal cortex **(G,H)** and medial entorhinal cortex **(I,J)****. Aci, anterior commissure intrabulbar; AOB, accessory olfactory bulb; cMEC, caudo-medial entorhinal cortex; CPu, caudatus putamen; dAON, anterior olfactory nucleus dorsal; LEC, lateral entorhinal cortex; lo, lateral olfactory tract; LV, lateral ventricle; MOB, main olfactory bulbs; OT, olfactory tubercle; PIR, piriform cortex; vAON, anterior olfactory nucleus ventral; VP, ventral pallidum. Calibration bar for **(A,C,E,G,I)** 500 μm.

Injections of RDA in the AON involved both dorsal and ventral components of the nucleus as well as the intrabulbar portion of the anterior commissure (Figures 2A,B). Injections in the OT mostly involved cell layer III including islands of Calleja (Figures 2C,D). Tracer deposits in the PIR were centered in cell layers II and III (Figures 2E,F). Similarly, injections in LEC mostly involved supragranular cell layers II and III (Figures 2G,H); whereas injections in MEC were centered in infragranular cell layers IV-VI (Figures 2I,J).

### Anterograde labeling

**Figure 3:**
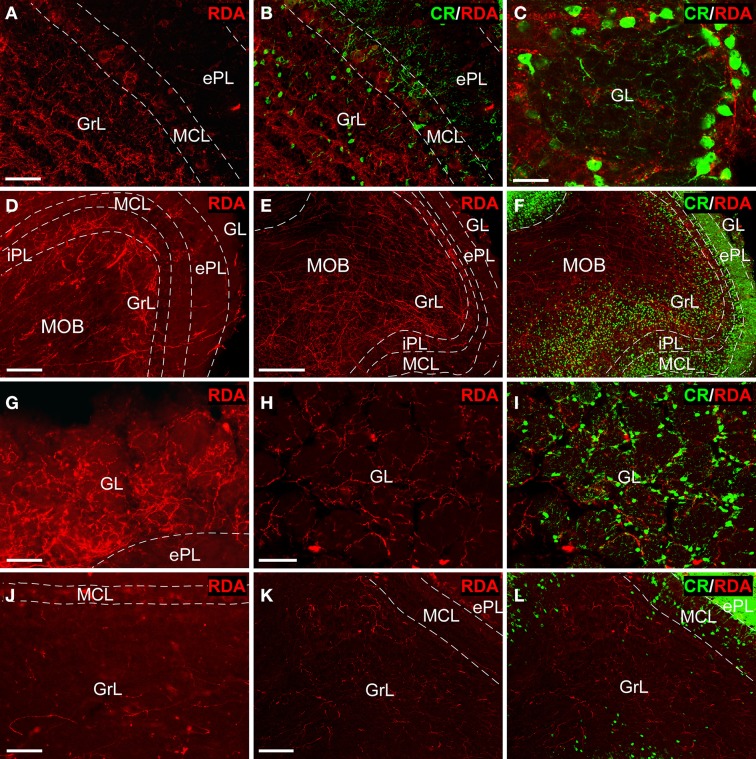
**Tract-tracing and immunofluorescence experiments**. Sagittal sections of the anterior olfactory bulb showing incoming rhodamine-labeled dextran-amine (RDA)-fibers from injections into the anterior olfactory nucleus **(A–C)**, olfactory tubercle **(D–I)** and piriform cortex **(J–L)** showing a rhodamine-labeled dextran-amine (RDA) under epifluorescence **(D,G,J)** and confocal **(A,B,C,E,F,H,I,K,L)** microscopy. CR, calretinin; ePL, external plexiform layer olfactory bulb; GL, glomerular layer olfactory bulb; GrL, granular layer; iPL, internal plexiform layer olfactory bulb; MCL, mitral cell layer; MOB, main olfactory bulbs. Calibration bar for **(A,B)** 25 μm; **C** 10 μm; **(D)** 100 μm; **(E,F)** 200 μm; **(G–L)** 50 μm.

Tracer injections in the AON (Figures 2A,B) resulted in anterogradely labeled fibers in the olfactory bulb. Confocal microscopy showed abundant, thin fibers concentrated in the granule cell layer (Figure 3A) as evidenced by double labeling with calretinin (CR) (Figure 3B). High-power images revealed labeled fibers around glomeruli in the glomerular layer (Figure 3C).

Injections in the OT (Figures 2C,D) yielded thick, labeled fibers under fluorescence (Figure 3D) and confocal (Figure 3E) microscopy throughout all layers of the OB as evidenced by CR expression (Figure 3F). Terminal-like fibers concentrated in the GL (Figure 3G) where they exhibited varicosities (Figure 3H). Double labeling experiments with CR demonstrated that most of these fibers were arranged in a periglomerular pattern (Figures 3C,I).

Tracer deposits in the PIR (Figures 2E,F) resulted in scarce, thin fibers mostly distributed into the GrL (Figure 3J). Confocal microscopy revealed that these fibers occasionally showed varicosities (Figure 3K). The preferential expression in the granule cell layer was assessed by CR immunoreactivity (Figure 3L).

**Figure 4:**
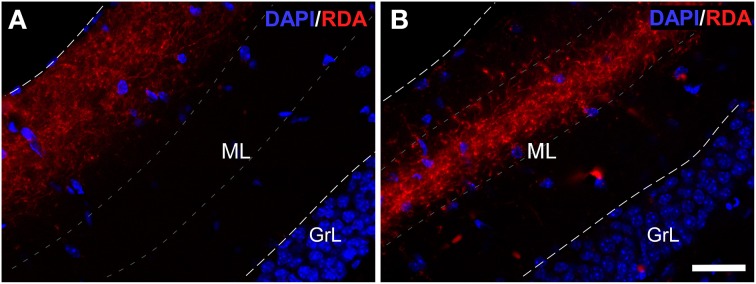
**Double immunofluorescence for DAPI (blue) and rhodamine-labeled dextran-amine (RDA) (red) showing lateral (LEC) and medial (MEC) entorhinal cortices**. LEC **(A)** and MEC **(B)** end in the outer and middle one-thirds of the molecular layer (ML) of the dentate gyrus, respectively. Calibration bar for **(A,B)** 25 μm. GrL: granular layer.

Experiments with RDA in the enthorinal cortex, aimed at the LEC (Figures 2G,H) and MEC (Figures 2I,J) labeled fibers along the perforant path that formed plexuses in the DG. Specifically, injections in LEC gave rise a band of labeled, varicose fibers in outer one-third of the molecular layer of the DG (Figure 4A); whereas injections in the MEC yielded a band of varicose fibers in the middle one-third of the molecular layer (Figure 4B). This labeling was only observed in all cases in the most caudoventral DG, from −3 to −4 mm posterior from bregma (Franklin and Paxinos, 2008).

### Immunofluorescence

**Figure 5:**
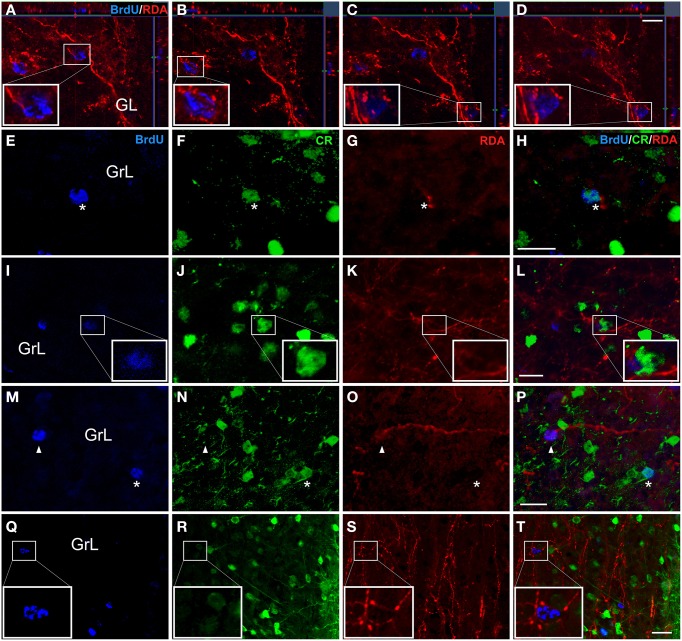
**Double and triple immunofluorescence againts bromodeoxyuridine (BrdU) (blue), calretinin (CR) (green) and rhodamine-labeled dextran-amine (RDA) (red)**. Asterisks indicate newly-born olfactory granule cells co-expressing BrdU and CR and establishing contacts with RDA-labeled terminal fibers from AON **(A-H)**, OT **(I-L)** and PIR **(M-T)**. Arrowheads show contacts between BrdU+ cells and RDA-labeled fibers. Calibration bar for **(A–D)** 10 μm, **(E–T)** 20 μm.

Confocal z-stacks images after injections in the AON (Figures 2A,B), showed BrdU-labeled nuclei in the GL surrounded by terminal fibers indicating presumptive axo-somatic synaptic contacts (Figures 5A–D). In the GrL, BrdU-labeled cells (Figure 5E), co-expressing CR (Figure 5F), were in close proximity to RDA-labeled fibers (Figure 5G), suggesting also putative axo-somatic contacts onto double labeled cells (Figure 5H). In experiments including RDA injections in the OT (Figures 2C,D), BrdU-labeled cells (Figure 5I) also expressed CR (Figure 5J) and RDA-labeled fibers (Figure 5K) suggesting contacts on the cell body of these cells (Figure 5L). In the cases with deposits in the PIR (Figures 2E,F), some BrdU-labeled cells (Figure 5M) co-expressed CR (asterisks in Figure 5N) and some others do not (arrowheads in Figure 5N). Fibers labeled with RDA (Figure 5O) appears to reach BrdU-labeled cells (arrowheads in Figure 5P) as well as BrdU- and CR-expressing cells (asterisk in Figure 5P). High magnification images demonstrated that 21 day-old BrdU-labeled cells (Figure 5Q), even not co-expressing CR (Figure 5R), were surrounded by labeled, beaded, terminal fibers (Figure 5S), establishing putative contacts (Figure 5T).

**Figure 6:**
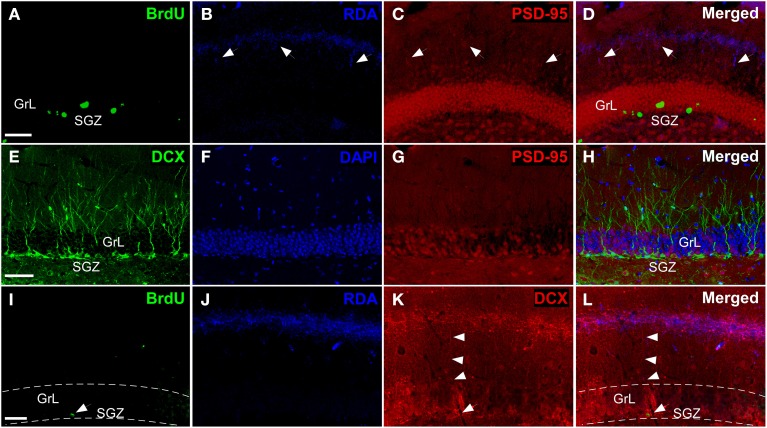
**Triple labeling experiments in dentate gyrus. (A–D)** Newly-born granule cells (green) were found in SGZ and arrows indicate connections between PSD-95+ cells (red) and rhodamine-labeled dextran-amine (RDA)-labeled fibers (blue) from cMEC. **(E–H)** Some of DCX+ cells (green), which are also localized in SGZ, express PSD-95 (red). **(I–L)** Arrowheads point out contacts between afferent RDA-labeled fibers (blue) from cMEC and BrdU (green)/DCX (red) double labeled cells. Calibration bar for **(A–C)** 50 μm.

Triple labeling experiments in the DG including injections in the entorhinal cortex combined with synaptic (Kosel et al., 1981) and maturational markers reveal hodological changes in newly-born neurons. Experiments including RDA injections in MEC (Figures 2I,J) reveal BrdU-positive cells in the SGZ (Figure 6A) and RDA-labeled fibers in the middle one-third of the molecular layer (Figure 6B). Interestingly, the expression of the postsynaptic marker PSD-95 (Figure 6C) matches that of RDA-labeled fibers (Figure 6D) thus suggesting presumptive synaptic contacts. The expression of doublecortin (DCX), a marker of neuroblasts during maturation and even maturation –up to 1 month after birth (Torubarova et al., 1981)-, shows the dendritic tree of young neurons occupying the full extent of the molecular layer (Figures 6E,F). Expression of PSD-95 (Figure 6G) (Kosel et al., 1981) suggests synaptic contacts on these cells (Figure 6H). Occasionally, BrdU-labeled cells (arrow in Figure 6I) and the resulting RDA labeling (Figure 6J) combined with DCX expression (arrowheads in Figure 6K) revealed BrdU-labeled cells (arrow in Figure 6L) displaying an apical dendrite (arrowheads in Figure 6L) reaching the zone of RDA-labeled fibers after injections in the MEC (Figure 6L). Because of BrdU protocol using HCl, immunodetection of some proteins, including PSD-95 and DCX becomes less sensitive as can be observed in Figures 6E vs. K.

In this sense, it is interesting to note that the length of the apical dendrites of DG granule cells -based on DCX expression and in agreement with previous studies (Torubarova et al., 1981)- is 95.5 μm ± 5.4 (s.e.m.) and 94.3 μm ± 6.9 (s.e.m.) in the caudoventral and rostrodorsal molecular layer of the DG, respectively. The outer and middle one-thirds of the molecular layer begin at 118.4 μm ± 9.8 (s.e.m.) and 74.2 μm ± 2.4 (s.e.m.) from the granule cell layer, respectively; which suggest that less 10% of the dendritic tree of DCX-positive granule cells reach the outer portion of the molecular layer.

## Discussion

**Figure 7:**
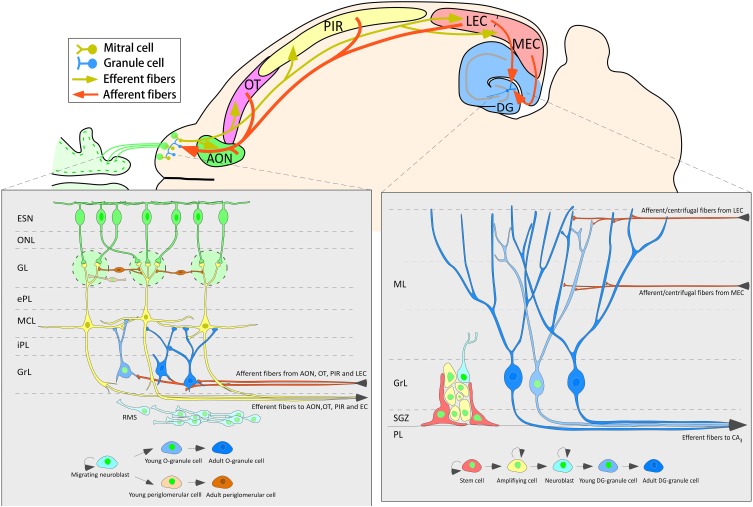
**Schematic diagram of one horizontal section of the mouse brain summarizing the neurogenic processes and the main afferent connections to the olfactory bulb and dentate gyrus**.

The data of the present work show that 3-week-old neurons born in the adult brain appear to receive inputs from distant structures. Newly-born granule and periglomerular cells in the OB, some of which co-express CR, could receive perisomatic inputs from axons of neurons located in the AON, OT or PIR (Figure 5). The centrifugal fibers, especially those originating in the AON and OT, appear to reach periglomerular cells (Figures 3C,G–I, 5A–D). Newly-born granule cells in the DG display apical dendrites over which axons from the MEC appear to make synapses in the middle one-third of the molecular layer. Although our results do not show it, the possibility that inputs from LEC reach the distal one-third of dendrites of newly-born cells cannot be discarded (Figure 7). Electron microscopy experiments have been carried out in order to further asses our results (see Supplementary Material), although results have been inconclusive due to technical difficulties.

### Adult-born granule cells in the adult olfactory bulb and dentate gyrus

It has been estimated that 30.000 cells per day are generated bilaterally in the mouse SVZ (Lois and Alvarez-Buylla, 1994); and that about 650.000 neuroblasts are present in granule cell layer of the OB 30 days after H^3^thymidine administration (Petreanu and Alvarez-Buylla, 2002). This process seems quantitatively less relevant in the DG, since the SGZ daily produce approximately 9000 and 1200 neuroblasts per day in rats and mice, respectively (Kempermann et al., 1997; Christie and Cameron, 2006). This totalizes about 0.1% of all DG cells (West et al., 1991; Rapp and Gallagher, 1996; Cameron and McKay, 2001). Approximately 50% of new-born cells survive and 80% of those become fully mature neurons (Dayer et al., 2003). More than 650.000 new granule cells are present in the hippocampus of 3-months old rats (Snyder and Cameron, 2012). However, in humans, a recent study measuring ^14^C concentration in neuronal nuclei found a high turnover rate in the human hippocampus, 700 new neurons are added in each side per day, corresponding to an annual turnover of 1.75% of the neurons. These rates are comparable in middle-aged human and mice, suggesting adult hippocampal neurogenesis plays an important role in the maintenance of brain function (Spalding et al., 2013).

SVZ mainly provides GABAergic granule and periglomerular new interneurons to the OB. Granule cells are the main population of axonless, GABAergic interneurons in the OB. These interneurons appear to modulate the activity of mitral and tufted cells optimizing olfactory function by reducing overlap of odor representation in these projection cells (Mori and Shepherd, 1994; Yokoi et al., 1995; Isaacson and Strowbridge, 1998). Granule cells receive both GABAergic and glutamatergic synaptic inputs from cortical areas (Price and Powell, 1970) and OB intrinsic neurons (Bovetti et al., 2011). Granule cells receive two functionally different glutamatergic synaptic inputs: mitral cells excite granule cells mainly through distal dendrodendritic synapses; whereas proximal smaller dendritic spines receive contacts from cortical neurons. Thus, these feedback inputs mainly coming from the AON, OT, PIR, and LEC (Anaya-Martinez et al., 2006; Mohedano-Moriano et al., 2012) can gate dendrodendritic inhibition (Balu et al., 2007).

Dentate granule cells are one of the most intriguing cells in the central nervous system since they are glutamatergic neurons, but also express, inter alia, GABA, suggesting that they can act as inhibitory cells (Gutierrez, 2003; Gutierrez and Heinemann, 2006). These cells have been demonstrated to be involved in certain learning and memory tasks (Toga and Lothman, 1983; Shors et al., 2001; Snyder et al., 2005; Deng et al., 2009) and in emotional and spatial behaviors (Saxe et al., 2006; Clelland et al., 2009; Goodman et al., 2010).

### Inputs onto adult-born granule neurons

Newly-born olfactory granule cells first receive GABAergic synaptic connections 3–7 days after birth, when the neuroblasts stop tangential migration and begin morphological maturation; and, 1 week thereafter, they begin to receive glutamatergic afferents (Belluzzi et al., 2003; Carleton et al., 2003; Panzanelli et al., 2009). Functional synapses at early stages of newly-born granule cells may establish a substrate for experience-depend regulation of adult neurogenesis and it can be important for long-term survival as data on blockade of glutamatergic activity have demonstrated (De Lima et al., 2004; Gascon et al., 2006; Panzanelli et al., 2009). Specifically, these centrifugal inputs may play an important role in maintenance and synaptic integration by regulation of dendritic growth of newly generated neurons in preexisting adult neuronal networks (Gascon et al., 2006; Mouret et al., 2009). However, knowledge about first synapses originating in neurons located in distant structures, including timing or specific origin is poor. In the present work, we have shown that olfactory afferents from AON, OT, and PIR reach and presumptively establish perisomatic contacts onto 21 day-old granule and periglomerular cells in the OB (Figure 5), even before they start to express specific olfactory population proteins.

Newly-born granule cells in the DG initially receive excitatory GABAergic inputs from local interneurons and this becomes inhibitory only once the glutamatergic extrinsic input from the EC has established, 2 weeks after birth (Overstreet Wadiche et al., 2005; Toni et al., 2007), showing most dendritic spines at 21–28 days after birth (Zhao et al., 2006). On the one hand, Li et al. (2012) have recently demonstrated that early GABAergic inputs may control the integration of maturing neurons to the adult hippocampal network. It has been shown that reduction of glutamatergic input implies loss of interneurons in other brain regions (De Lima et al., 2004). Many studies reveal the importance of entorhinal inputs to dentate granule cells. For example, unilateral or bilateral olfactory bulbectomy implies a rapid, transient increase in apoptosis in the dentate gyrus ipsilateral to the removed OB and lesions of the MEC significantly increase the number of dividing cells in the dentate gyrus in adult rats (Pope and Wilson, 2007). Data suggest that afferent input by NMDA receptor activation slows neuronal birth during adulthood (Cameron et al., 1995). Thereby, inputs seem to play a relevant role for cell survival, maturation and integration of new dentate granule cells once they reach maturity.

Dentate granule cells receive, through the perforant path, massive afferents from LEC and MEC (Steward, 1976; Witter, 2007). This pathway uses glutamate as primary neurotransmitter via NMDA receptors (White et al., 1977; Collingridge, 1989) but also presents a small GABAergic population (Germroth et al., 1989). Interestingly, neurons located in LEC send apical dendrites to layer I where they receive afferents from axons of bulbar mitral and tufted cells (Kosel et al., 1981; Martinez-Marcos and Halpern, 2006; Martinez-Marcos, 2009). Neurons located in layers II, V, and VI of LEC send axons to the outer one-third of the molecular layer of the DG; whereas the corresponding cells of MEC establish synapse in the middle one-third of this layer (Ramón Y Cajal, 1893; Hjorth-Simonsen, 1972; Hjorth-Simonsen and Jeune, 1972) (Figure 6). The dentate gyrus, therefore, receives olfactory information through the entorhinal cortex (Kosel et al., 1981) and the ventral subiculum and CA1 projects back to the OB (De La Rosa-Prieto et al., 2009b; Mohedano-Moriano et al., 2012). These data suggest that dentate granule cells could be also involved in olfactory processing, specifically in pattern separation for olfactory learning (Gilbert et al., 2001; Kesner et al., 2011), odor discrimination (Eichenbaum et al., 1988) and olfactory memory (Staubli et al., 1984).

### Conflict of interest statement

The Associate Editor Jose Manuel Garcia-Verdugo and the Review Editor Francisco E. Olucha-Bordonau declare that, despite being affiliated to the same institution as authors Teresa Liberia and Carlos Crespo, the review process was handled objectively and no conflict of interest exists. The authors declare that the research was conducted in the absence of any commercial or financial relationships that could be construed as a potential conflict of interest.

## Abbreviations

AON: anterior olfactory nucleus
BrdU: bromodeoxyuridine
CR: calretinin
DCX: doublecortin
DG: dentate gyrus
EC: entorhinal cortex
GL: glomerular layer
GrL: granular cell layer
LEC: lateral entorhinal cortex
MEC: medial entorhinal cortex
OB: olfactory bulb
PB: phosphate buffer
PBS: phosphate buffered saline
PIR: piriform cortex
PSD-95: postsynaptic density-95
RDA: rhodamine-labeled dextran-amine
SGZ: subgranular zone
SVZ: subventricular zone
TBS: Tris buffered saline.
