# Supplementary material for: Olfactory and cortical projections to bulbar and hippocampal adult-born neurons
Source: Front Neuroanat. 2015 Feb 2;9:4. doi: 10.3389/fnana.2015.00004 (PMC4313705; doi:10.3389/fnana.2015.00004)

**SUPPLEMENTARY MATERIAL**

In order to further asses the establishment of synaptic contacts onto newly-born cells, electron microscopy experiments were carried out. Six additional mice received intraperitoneal BrdU injections and biotinylated-labeled dextran-amine (BDA) in the anterior olfactory nucleus (n=2), olfactory tubercle (n=2) and piriform cortex (n=2). These animals were perfused with 4% paraformaldehyde and 0.5% glutaraldehyde in 0.1 M phosphate buffer (PB).

## Material and Method

### Tracer detection and BrdU immunohistochemistry for electron microscopy

Biotinylated dextran amine (BDA) was visualized by using the avidin-biotin-peroxydase reaction as follows: First, sections were rinsed in 0,1 M PB (3x10 min), cryoprotected by 20 min immersion in a mixture of 25% sucrose and 10% glicerol in 0,01 M PB and freeze-thawed three times with liquid nitrogen, in order to enhance Avidin-biotinylated horseradish peroxidase complex (ABC; Vector Labs. Burlingame, CA, USA) and later antibody penetration. Then, all sections were incubated in ABC, diluted 1:200 in PB, for 2h at room temperature and carefully rinsed in 0,1 M PB (3x10 min). Finally, the peroxidase reaction was developed using 0.05% 3,3-diaminobenzidine tetrahydrochloride (DAB, Sigma-Aldrich, St. Louis, MO, USA) as chromogen and 0.003% hydrogen peroxide in PB, for 5 min at room temperature until labeled fibers could be visualized under light microscopy. Then, sections were rinsed in PB (3x10 min) and the immunohistochemistry for the detection of BrdU was performed.

BDA-containing sections were treated with 1% NaBH4 in PB for 20 min and extensively rinsed in 0,1M PB. Then, they were incubated in 2 N HCl in 0,1 M PB for 30 min at room temperature in order to denature DNA and carefully rinsed in PB. The immunocytochemical detection of BrdU was performed using the ABC method as follows. Sections were sequentially incubated in: (a) blocking solution, containing 10% normal donkey serum (NDS) and 0.05% sodium azide in PB, for 60 min at room temperature. (b) Rat anti-BrdU antibody (1:1,000 Ascites ) diluted in PB containing 1% NDS and 0.05% sodium azide, for 48h at 4ºC. (c) Biotinylated donkey anti-rat igG (Jackson ImmunoResearch Laboratories, PA; USA) diluted 1:200 in PB, for 2h at room temperature. (d) ABC diluted 1:200 in PB, for 2h at room temperature. After each step, sections were carefully rinsed in PB (3x10 min). Finally, the peroxidase reaction was developed using 0.05% DAB as chromogen and 0.003% hydrogen peroxide in PB, for 5 min at room temperature, until specific BrdU-immunostaining could be visualized under light microscopy. Then, sections were carefully rinsed in 0,1 M PB (3x10 min), treated with 1% osmium tetroxide (Electron Microscopy Sciences, Hatfield, PA, USA) containing 7% glucose in PB for 45 min at room temperature and extensively washed in PB. Finally, sections were stained with 1% uranyl acetate (Electron Microscopy Sciences) in maleate buffer, pH 4.5 for 90 min, dehydrated through graded ethanol series, cleared in propylene oxide and flat-embedded in Durcupan (ACM, Fluka AG, Switzerland) between slides and coverslips. Durcupan was polymerized overnight at 60ºC. Flat-embedded sections were carefully examined at the light microscope and some fields where BrdU-positive cells and tracer-labeled fibers were juxtaposed were selected and re-embedded in Durcupan for further analyses under electron

microscopy. The selected sections were cut on an ultramicrotome, and serial 60-nm-thick ultrathin sections were obtained and mounted on single-slot Formvar-coated nickel grids. The ultrathin sections were stained with lead citrate and analyzed at the electron microscope.

Controls were carried out by omitting the first and second antibodies in each step and by incubating some sections exclusively in 0.05% DAB and 0.003% hydrogen peroxide in PB in order to exclude the presence of endogenous peroxidase in the tissue and assess the specificity of the immunohistochemical method. No residual activity was found in these controls.

**LIGHT MICROSCOPY**

Supplementary figure 1

Semithin sections illustrate some boutons that apparently innervated BrdU-containing cells under light microcopy (arrows). Scale bar 10 µm.

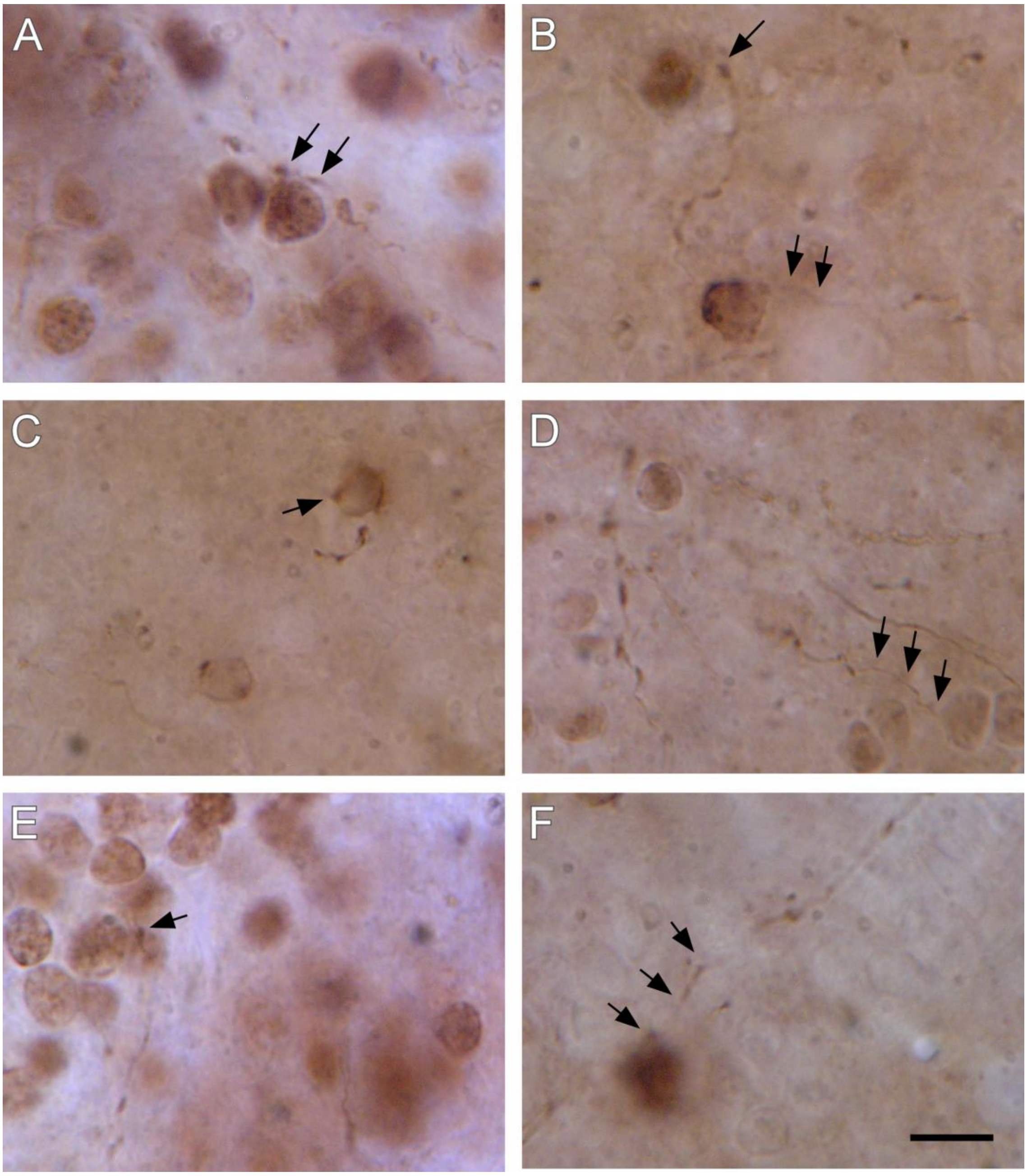

<u>Supplementary figure 2</u>

High power magnificacionts illustrate some boutons in semithin sections that apparently innervated BrdU-containing cells under light microcopy (arrows). Scale bar 10 µm.

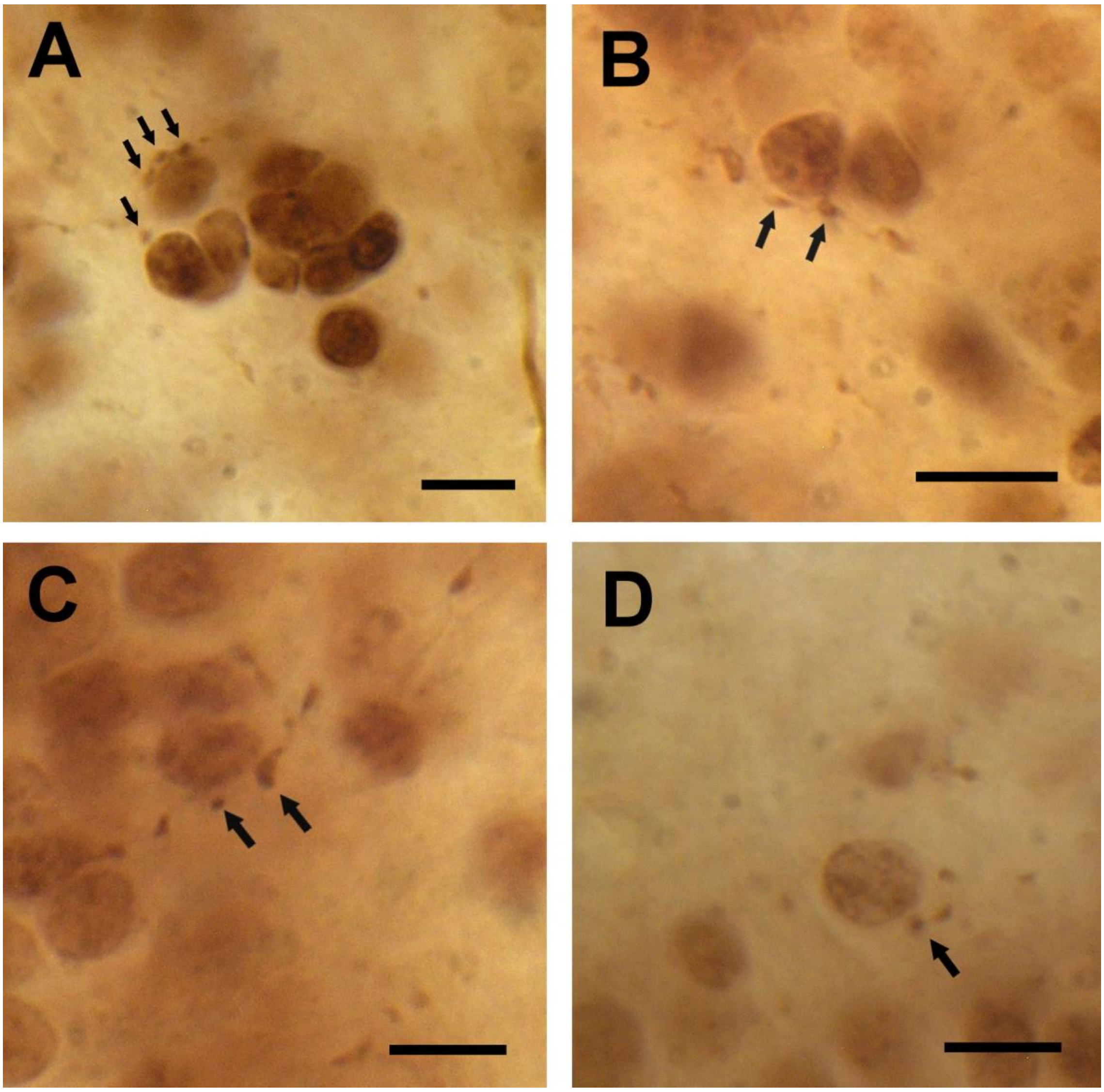

Supplementary figure 3

The BrdU-immunopositive cells (asterisks) and the tracer-containing axons (arrows) were identified. Unfortunately, the ultrastructure of the tissue is poorly conserved because of the HCl-treatment. Scale bar for A, B and E, 1 µm. C,D, 800nm and F, G and H 2µm.

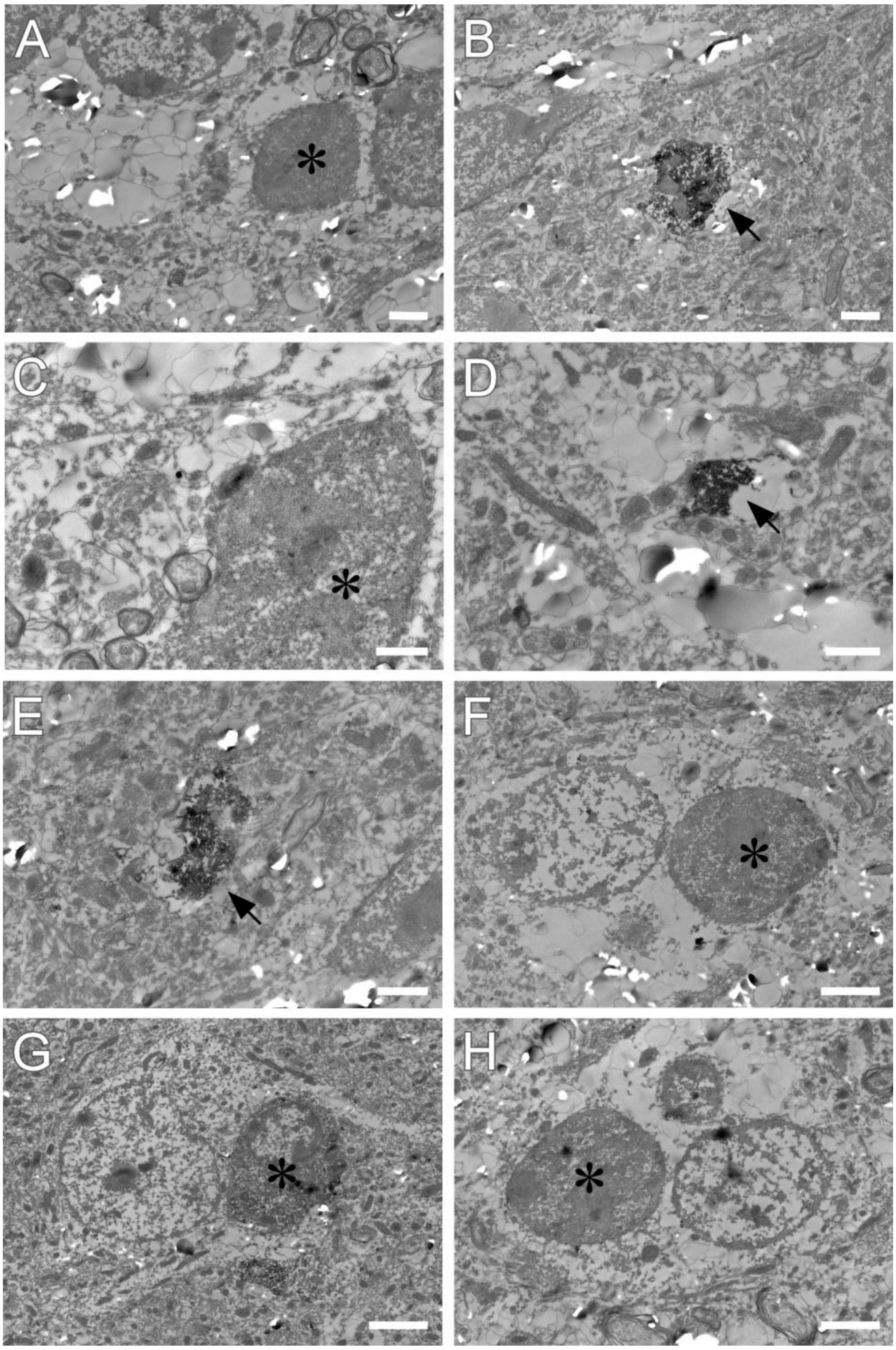

Supplementary figure 4

Note that the neuropil surrounding two BrdU-containing cells (A-B) and two tracer-containing axons (C-D) is severely destroyed (asterisks). The poor conservation of the ultrastructure after BrdU-immunocytochemistry does not allow us to analyze and identify the presence of synaptic contacts. Scale bar for A-B, 1µm and C-D, 800nm

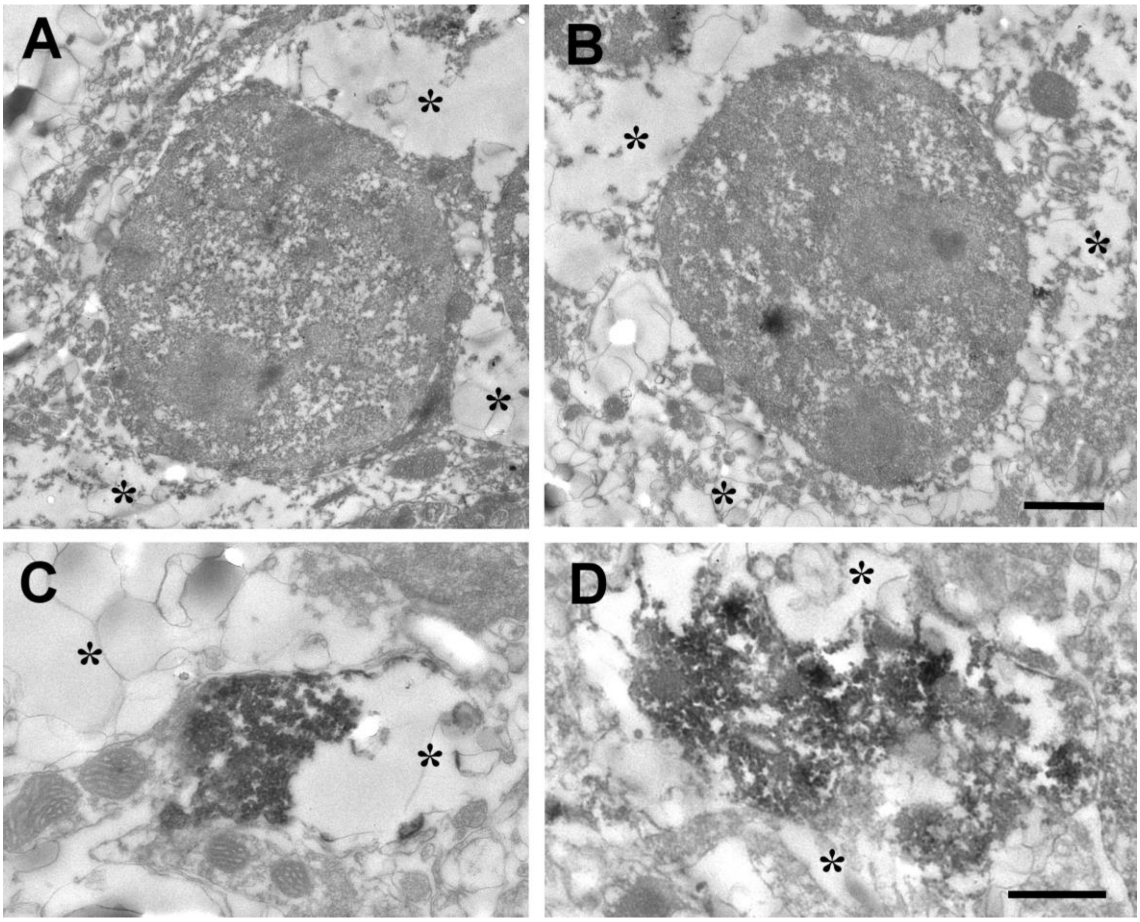

Supplement: Supplementary file 1 [file Image1.PDF]
